# Mating system and copulatory behavior of the greater mouse‐eared bat (*Myotis myotis*)

**DOI:** 10.1111/nyas.15390

**Published:** 2025-06-18

**Authors:** Lisa Printz, Anika Lustig, Martina Nagy, Mirjam Knörnschild

**Affiliations:** ^1^ Museum für Naturkunde – Leibniz Institute for Evolution and Biodiversity Science Berlin Germany; ^2^ Institute of Biology, Freie Universität Berlin Berlin Germany; ^3^ Faculty for Biology Ludwig‐Maximilians‐Universität München München Germany; ^4^ Koordinationsstelle für Fledermausschutz Südbayern München Germany; ^5^ Institute for Biology Humboldt‐Universität zu Berlin Berlin Germany; ^6^ Deutsche Fledermauswarte e.V. Berlin Germany

**Keywords:** copulatory behavior, European bats, lek mating system, mating behavior, territorial behavior

## Abstract

Mammalian mating systems, which form the cornerstone of social systems, are shaped by diverse ecological and sociobiological factors, and they influence behavior and reproductive success. Among mammals, bats exhibit a remarkable diversity of mating systems, making them ideal for studying their complexity; yet, interspecific variations of bat mating systems remain largely unknown. To address this, we surveyed six roosts of the greater mouse‐eared bat (*Myotis myotis*) over 2 years, uncovering novel aspects of their mating system. Our findings suggest a lek mating system, where males aggregate and are visited by receptive females. Mating involves multiple copulations and distinct body postures, with the female remaining with the male for several hours. Male roost occupancy peaked in August, reflecting a phenological cycle. Males demonstrated pronounced territoriality and site fidelity, defending display spots with vocalizations and physical confrontations, underscoring their important role in securing mating success. Complex vocalizations appeared crucial for deterring rivals and attracting females, suggesting vocal signals govern mate choice by females. Additionally, a yellow facial secretion observed in males may function as an olfactory signal during mate selection. This study provides valuable insights into the mating system of *M. myotis*, with implications for understanding the species’ behavioral ecology and contributing to conservation strategies.

## Graphical abstract

The greater mouse‐eared bat (*Myotis myotis*) likely exhibits a lek mating system, where males aggregate at specific sites and are visited by females during the mating season. Throughout the season, males show high territoriality and display spot fidelity and emit conspicuous vocalizations that potentially impact female mate choice. Mating is characterized by distinct body positions and involves multiple copulations, with females remaining for hours on the male display spot.

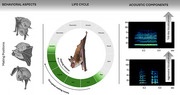

## INTRODUCTION

Animal mating systems are diverse and play a central role in evolutionary biology. It is, therefore, important to describe how females and males mate and reproduce successfully.^1^, ^2^ Explaining the evolution of these adaptations is a major challenge due to the vast array of traits, behaviors, and strategies involved. In mammals, mating systems include monogamy, polygyny, polyandry, promiscuity, and lek mating, with several subcategories. Mating systems are affected by a variety of factors (e.g., environmental factors, philopatry, dispersal), with sexual selection influencing secondary sexual traits and conflicts.^2^, ^3^, ^4^ Thus, understanding mating systems is essential for understanding the fundamental behavioral ecology of a species and enabling effective species management and conservation efforts.

Bats exhibit a diverse array of mating systems, yet a recent review showed that the mating systems of only 6% of bat species have been described.^5^ There have been several attempts to categorize mating systems,^1^, ^6^, ^7^ but the observed diversity occurs along a continuum, thus making categorization difficult. Furthermore, mating systems are commonly perceived as inherent species traits, but there is increasing evidence for variability even within species.^3^, ^8^

Following the redefinition of Dorrestein et al.,^5^ bat mating systems can be divided into seven categories, including monogamy, promiscuity, female defense polygyny, resource defense polygyny, and three classes of lek mating systems. However, when investigating animal mating systems, it is essential to differentiate between social and genetic mating systems.^9^ Social mating systems reflect the observed structure of mating relationships, such as resource defense polygyny, where a single male appears to monopolize access to several females by controlling resources.^5^ Genetic analyses often reveal a different pattern: when resources are abundant and females can move freely, they frequently mate outside their social group since there is no benefit of staying in one particular place.^10^, ^11^ Consequently, while the social system may appear polygynous, the genetic system is often polygynandrous, with females mating with multiple males. The concept of male reproductive skew effectively illustrates these systems as a continuum, highlighting that mating structures do not fit into rigid categories but rather exist on a fluid spectrum where genetic and social structures intersect.^5^

A small number of bat species is known to be monogamous.^5^ Monogamy, characterized by one male exclusively mating with a single female, suggests a high level of male investment in female and offspring survival. While monogamy has been seen as the predominant mating system in birds,^12^ it is comparatively uncommon among mammals.^3^, ^13^ However, there are several bat species that are monogamous.^6^, ^14^ Here, individuals form long‐term pair bonds and share parental care. This mating system is particularly prevalent in species with stable social structures and limited opportunities for extra‐pair copulations. In contrast, promiscuous species do not form continuing pair‐bonds and mate several times with different mating partners.^3^, ^5^ Most bat species are polygamous, which is defined as a mating system where one or both sexes engage in mating with multiple partners.^5^ In polygamous systems, males adopt various strategies to monopolize mating access to females. Males either defend multiple females directly (i.e., female‐defense polygyny) or they defend resources essential to females such as foraging or roost sites. This competition often results in territoriality among males, with dominant individuals securing preferential access to females and a mating system termed resource‐defense polygyny. When neither females nor resources can be economically defended, males may establish resource‐free mating territories, so‐called leks.^3^ The lek mating system is characterized by the central role of female choice. Substantial energy is invested in attracting females by producing sexual displays comprising visual, acoustic, and olfactory signals, either individually or in combination. Receptive females visit the males aggregating on these sites solely to select males for mating.^5^, ^15^ The females can move freely between the males and do not get any direct benefits from the males. However, the male advertising behavior functions as a proxy for male quality. Despite being the focus of research for many years, this mating system is still poorly understood in bats.^15^

Among all mammals, individual male behavior plays a crucial role in determining mating success, and the way individuals interact (e.g., mate selection, aggressive interactions) directly influences their chances of reproductive success.^16^ However, these behaviors can vary significantly depending on the mating system. In monogamous mating systems, male behavior mainly focuses on parental care, assisting in births, and protecting the young,^17^ contributing to higher survival rates of the young and greater breeding frequency of the females.^18^ The aggregation of multiple females often exerts strong selective pressure on males, leading to the development of traits that enhance their competitive abilities.^16^ This is frequently associated with the evolution of sexual dimorphism and weaponry, particularly in species with seasonal breeding.^17^ One common male behavior among different mating systems is aggressive behavior, which can be observed in female‐defense polygyny, resource‐defense polygyny, and lek mating systems.^5^, ^16^ In addition to the aggressive behavior exhibited by males to defend established territories, courtship is an important behavioral trait and can be seen in lek mating systems.^19^

Vocal courtship displays have been described for several bat species.^20^ This indicates that male bats have evolved vocalizations to attract mates and/or to defend their territories against other bats. Since choosing the right mate can strongly affect the fitness of an individual,^21^ complex courtship behavior allows females to assess the quality of potential mates, such as physical condition and genetic compatibility.^22^ Overall, courtship behavior in bats is highly variable and can include tactile (e.g., mutual grooming^23^), visual (e.g., wing flapping^24^), olfactory,^25^ or acoustic components,^20^ often presented in a multimodal way. Furthermore, courtship behavior, along with aggressive behavior, is decisive for the mating success of a male.^16^ Courtship activity can occur in the bats’ day‐roost,^26^ specifically in temporal mating roosts^27^ or designated swarming sites.^28^ Despite knowledge of exceptional aspects of bat reproduction (e.g., prolonged sperm storage^29^), many basic aspects of bat mating systems and associated male behavior remain unknown, largely due to the elusive lifestyle of bats.^7^ One cornerstone of reproductive behavior is copulatory behavior, which has been to date only described for a few bat species;^30^, ^31^, ^32^ again, exhibiting unique copulatory behaviors among mammals, such as mating without penetration.^33^ Since the mating behavior of bats is seldomly observed in captivity, it is necessary to directly observe social interactions of bats in their natural roosts, but these are in turn often hardly accessible.

*Myotis myotis*, the greater mouse‐eared bat, is a large insectivorous bat found across Europe and parts of Asia.^34^, ^35^ Understanding its behavioral ecology, including the mating behavior, is crucial for effective conservation, particularly in addressing challenges such as roost disturbances. Current conservation practices often assume that male bats are more adaptable than females and less loyal to existing roosts. Consequently, building renovations affecting male roosts are frequently permitted by local nature conservation authorities without strict seasonal restrictions. *M. myotis* follows an annual triphasic activity cycle typical of temperate‐region bats.^35^ In winter, both sexes hibernate in mixed clusters.^36^ After hibernation, males and females separate; females form nursery roosts during parturition and lactation,^35^, ^37^ while males roost alone.^34^, ^38^ Little is known about their mating system and male social behavior during this segregation. Anecdotal observations indicate that male *M. myotis* defend their roosting site against other males and show territorial behavior.^34^, ^38^, ^39^, ^40^ During mating and courtship behavior, two types of male vocalizations and distinctive body postures before and after copulation have been described,^38^ but their function remains unknown.

In this study, we investigated the behavior of male *M. myotis* during the mating season, as the full range of male behavior has not been described previously. Our aim was to categorize the mating system of *M. myotis* based on observations during our study period. Based on prior observations,^38^, ^40^ we propose that (1) males defend territories through aggressive interactions during the mating season from August to October. Furthermore, we expected to find characteristic copulatory behavior and body postures, as observed by Zahn and Dippel.^38^ We hypothesized that (2) distinct behavioral patterns would emerge in males during the mating season, including pronounced display behavior. Additionally, we expected that (3) male roost occupation would follow a distinct seasonal pattern, with males occupying roosts during specific times of the season. In addition to providing new insights into the behavioral ecology and the mating system of *M. myotis*, we emphasize the critical need to preserve or replicate males’ display sites at their original locations following restoration activities, ensuring the continuity of mating roost functionality.

## METHODS

### Study site

**FIGURE 1:**
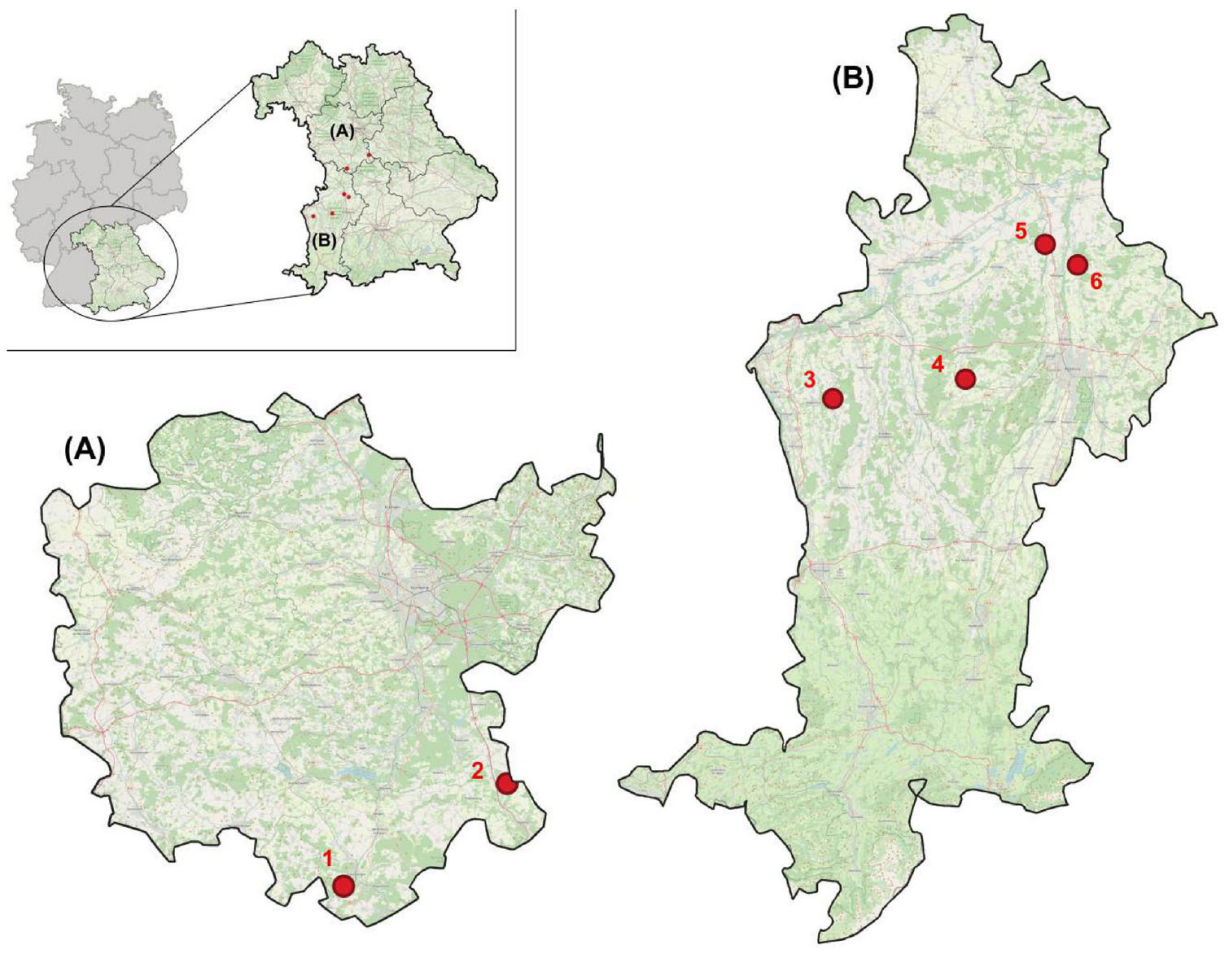
Map of the study area (openstreetmap.org/copyright). All roosts are located in the federal state of Bavaria, Southern Germany, and are spread across the two administrative districts of (A) Middle Franconia and (B) Swabia. Red dots indicate the location of the selected male roost used for our observational study.

*M. myotis* is a perennial cave‐dweller in southern Europe but depends on buildings as roosts (e.g., attics, churches, or castles) located north of the Alps.^34^, ^35^, ^41^ Similar to natural cave roosts, these spaces are relatively unstructured and offer a variety of retreat options. The greater mouse‐eared bat is well‐suited for behavioral studies due to its fidelity to roosting sites and its preference for free hanging inside the roosts. Our study was conducted in Bavaria (Figure 1), Germany, during two consecutive summers (May−October 2022 and 2023). We defined a male roost as a roost that is frequented exclusively by males during summer.

All inspected roosts were located in church towers or large attics. Out of 76 inspected roosts, we selected six different male roosts of *M. myotis* based on their suitability for behavioral observations. Each roost was inhabited by at least three males (Table S1), featured the characteristic discolorations on display spots within the roost (caused by urine and grease from the fur), and was securely accessible for observations. Before the investigations began, all bats (*N* = 73) were habituated to the presence of human observers, enabling us to investigate their natural behavior and to record associated vocalizations without disturbance. Additionally, we made schematic maps of the location of the stained display spots for each selected roost and documented changes in the number of bats per roost at least twice a week. We also documented any changes in display spot occupancy for 17 males inside the respective roosts in 2023, based on either distinctive visual characteristics (e.g., scars), or by a unique pattern on the back of the bat which we created by trimming the fur. Accessing and entering the bat roosts was approved by the associated conservation authorities. All field work and experimental protocols were approved by the German authorities (Schwaben 55.1‐8646‐2/1148; RMF‐SG55.1‐8646‐10‐28‐2) and the responsible organizations for bat protection (Coordination office for bat protection, Northern and Southern Bavaria).

### Behavioral observations

Each male roost was regularly visited for at least one season, starting from May to the beginning of October in 2022 and 2023 (Table S1). The starting point of our field season was determined by the regular presence of males inside the roosts, which was at the end of May. We ended the field season when the males had left the roost, or when a large number of females had started to gather inside, as this was an indication for all bats leaving in the following days. As all the roosts were either in churches or privately owned buildings, the dates for recording sessions depended on when the owners of the buildings granted us access. Thus, there are fluctuations in the frequency of roost inspections.

Behavioral observations were conducted during the mating season, typically occurring between August and October.^34^ Observations were carried out during the night as well as during the day. An observation and recording session lasted up to 9 h, depending on the activity of the bats on the respective date. We used infrared light sources to illuminate the roosts without disturbing the bats.^42^ In addition to the in‐person observations and for subsequent context analysis, a digital camcorder with a night‐shot function was left running (Sony DCR‐SR). Several behavioral variables were recorded to assess male behavior, including descriptive observations of male courtship displays, mating events including copulation behavior, aggressive behaviors between males and other individuals, and female mate choice behavior. We defined a mating event as an occurrence of reproductive behavior between two individuals; it may include successful copulation(s) or solely mating attempts. A mating event begins when a female arrives at a male display spot and is considered complete once she left the male. However, we faced limitations in determining how often individual males copulated throughout the mating season as we could not monitor the males continuously. We focused on male behavior; thus, female presence was not systematically assessed.

To monitor the social situation inside the male roosts and document mating events in their entirety, we set up ultrasonic recording devices (Avisoft USG 116Hme, with condenser microphone CM16; frequency range 1–200 kHz, sampling rate 500 kHz, 16‐bit depth resolution) connected to a laptop computer running the software Avisoft Recorder (v4.2.05, R. Specht, Avisoft Bioacoustics). To comprehensively cover the season, we additionally set up passive recording devices inside the roosts (Song Meter Mini Bat, sampling rate 384 kHz, 16‐bit depth resolution, Wildlife Acoustics). To unambiguously assign the recorded vocalizations to one individual during a mating event, we used a sound camera (SoundCam Ultra IR, CAE Software and Systems GmbH). The sound camera visualizes sound and superimposes this image synchronously over the video recording, allowing us to visually confirm the vocalizing individual.

### Analysis

Throughout the 2‐year study period, we made behavioral observations for a total of 755 h distributed across 195 inspection days in six roosts. Within this timeframe, we meticulously documented the presence and behaviors of 73 individual males within the selected roosts (Table 1). Among those observations, we also noticed 36 mating events of *M. myotis*. We focused on successful mating events (*N* = 27) and did not include any instances of attempted or interrupted mating (*N* = 9).

**TABLE 1:** Ethogram including definition for all behaviors.

Behavior	Description
**Sleeping/resting**	No movement, motionless, no vocalizations.
**Self‐grooming**	Licking/nibbling fur and wings, scratching with feet, no vocalizations.
**Scanning of surroundings**	Lifting head up, mouth open, looking around calmly, body stays calm, echolocation calls.
**Wing stretching**	Bat opens one/both wings completely, no vocalization.
**Displaying**	Single male roosting at display spot; despite there being no disturbance inside or near roost, the head is raised and the mouth slightly opened, complex vocalizations are produced.
**Aggression (when a male is landing near the display spot of another male)**	Biting and hitting with wing(s), crawling toward other bat, producing aggressive vocalizations.
**Aggression (when a bat is flying around)**	Head is raised, line of sight follows the bat flying around, crawling around on display spot, aggressive vocalizations.
**Copulation**	Male roosts with its belly on the back of a female, immobilizing the female with its wings; several copulations, rhythmic movement of the lower abdomen.
**Male grooming female**	Male turns its head to lick the female in the mouth or neck area, either while the male roosts on the back of the female or vice versa.
**Female grooming male**	Only during mating. Female licks and nibbles male.
**Male resting on the back of female**	Male rests motionless on the back of the female. Female slightly opens her wings.
**Male clasping female**	Male roosting belly‐to‐back with female and clasping her with his wings completely. Often before and during copulation.
**Females roosting on display site with one male**	One or two female bats resting in the male display area, very calm, no aggressive behavior.

*Note*: This ethogram provides a comprehensive catalog of social behaviors observed in male roosts of *Myotis myotis* over a 2‐year period. The behaviors were systematically categorized based on in‐person observations and recordings, all in a mating context.

We analyzed all video recordings and in‐person observations manually, focusing on identifying mating events. Through close examination of interactions between bats and their associated behaviors, we generated an ethogram (Table 1). Additionally, we analyzed the frequency of complex, trill‐like vocalizations in recordings from two roosts (roost 3 and 4, total amount of recordings *N* = 16,953) that we were able to acoustically monitor continuously throughout the 2023 season using Song Meter Mini Bat recorders (Wildlife Acoustics). The template scanning function in Avisoft SASLab Pro (version 5.2) was used to detect these trill‐like vocalizations. The detection process relied on preselected templates of representative vocalizations. To enhance the accuracy of call detection, background noise was reduced by applying a band‐pass filter to eliminate frequencies below 12 kHz and above 80 kHz. We then analyzed the occurrence frequency of these vocalizations throughout the season. Therefore, we fitted a negative binomial regression model with a log link function to assess the relationship between the total number of trills and the month as ordered factors, including a random effect for roost (R package *glmmTMB* ^43^). This approach was chosen to address overdispersion observed in our data and was further validated by post‐hoc analyses (estimated marginal means [EMMs] and Tukey method; R package *emmeans* ^44^). Moreover, we analyzed the timing of male bats being present inside male roosts over the course of each study season, aiming to gain insights into the phenology of male roost occupancy in *M. myotis*. To assess the statistical significance of differences in male bat occupancy across months, we fitted a generalized linear mixed model (GLMM; R package *lme4* ^45^) with month as an ordered factor and using a Poisson error distribution. The model included a random intercept for each roost site to account for variability among locations. To evaluate pairwise differences in male numbers across months, we conducted a post hoc analysis, again using EMMs from the fitted GLMM and the Tukey method to adjust for multiple testing (R package *emmeans* ^44^). All statistical tests were performed in R Studio, with a significance level set at *p* < 0.05.

## RESULTS

**FIGURE 2:**
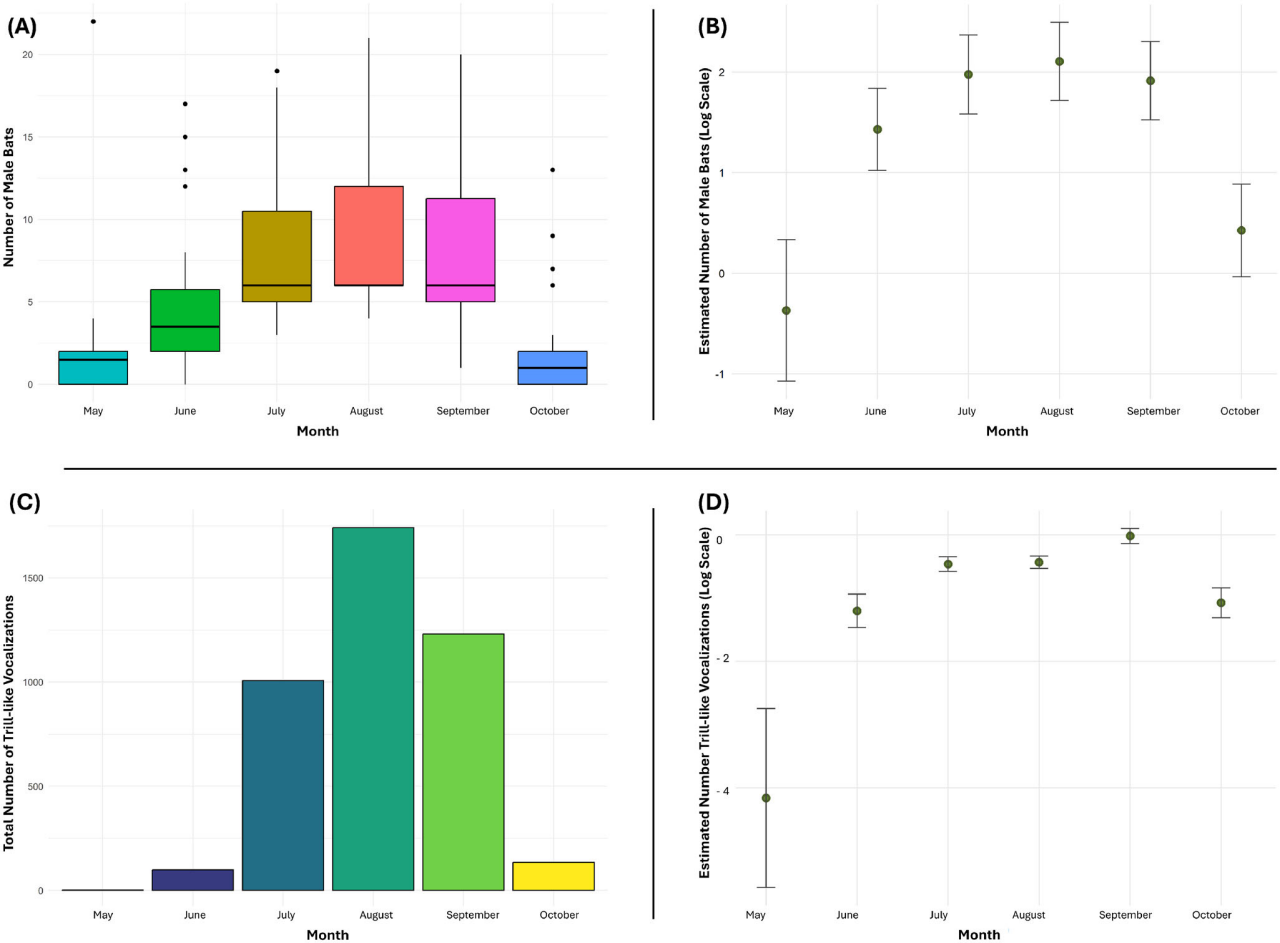
Number of male bats across the season. (A) Summed number of male bats encountered inside the selected roosts per month over the 2‐year study period. (B) Estimated monthly mean number of male bats with 95% confidence intervals. (C) Total number of trill‐like vocalizations detected in recordings during the long‐term monitoring of two roosts in 2023. (D) Estimated monthly mean number of trill‐like vocalizations with 95% confidence intervals. Green dots represent the estimated mean values based on the statistical model, while the vertical lines (error bars) indicate the uncertainty range for each estimate. Male bat activity peaks during the summer months (July−September) and declines in October, with greater variability observed in May and October.

**FIGURE 3:**
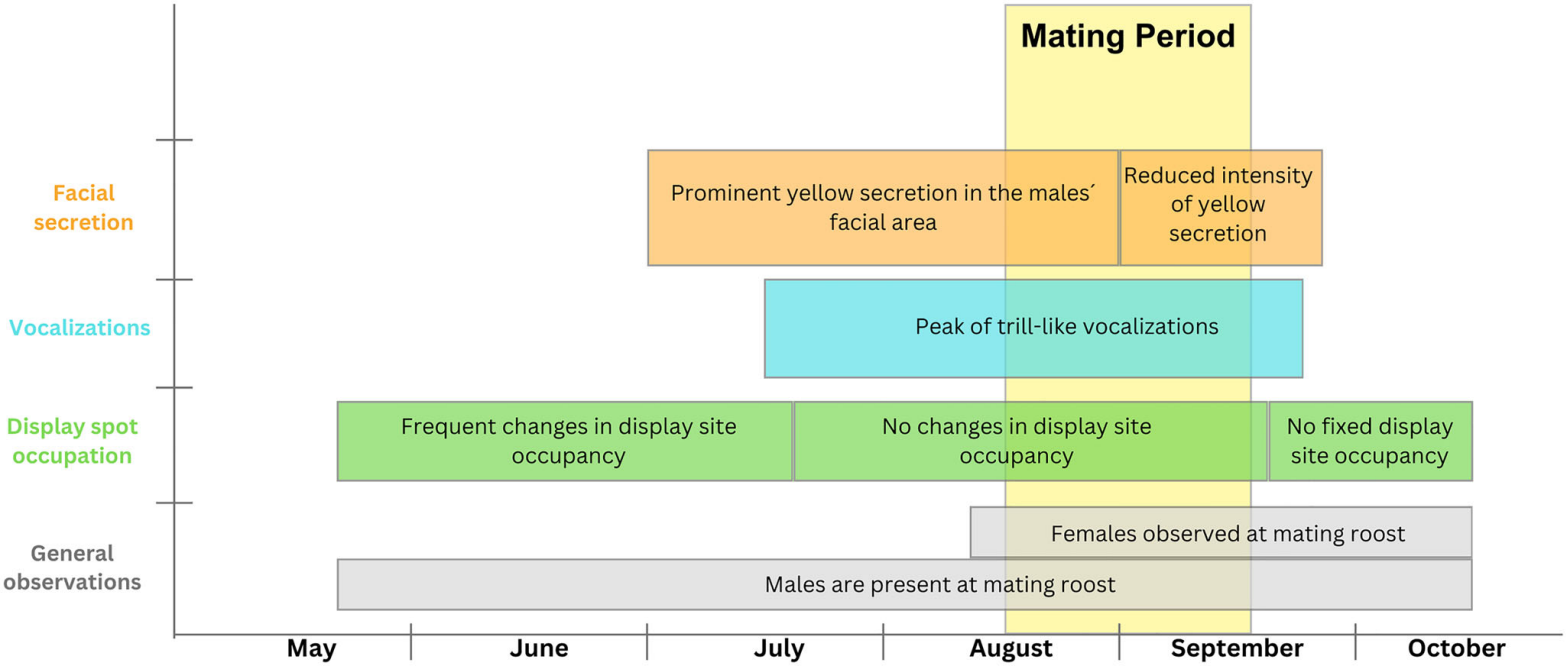
Phenology of male traits and behaviors observed at roosts across the season. The timeline summarizes the seasonal patterns of facial secretion, vocalizations, display site occupation, and general observations. The mating period is highlighted in light yellow.

Throughout the study period, the occupancy of male roosts exhibited dynamic patterns and the number of male bats varied significantly among the months (Figure 2A). The number of male bats showed a consistent rise month by month (estimate = 0.6638, *p* < 0.001). This pattern was not purely linear; a significant negative quadratic effect (estimate: −2.1160, *p* < 0.001) suggests a curvilinear shape, peaking in August (Table S2). Starting from mid‐September, male bats gradually left the roosts and the number of bats declined, resulting in vacant roosts by the end of October. These results highlight the seasonal trend of male bat occupancy and clearly show a complex pattern in male bat occupancy across months (Figures 2B and 3).

**FIGURE 4:**
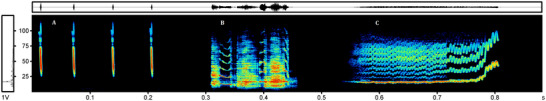
Vocalizations recorded inside male roosts. (A) Echolocation calls. (B) Aggressive interactions between males. (C) Complex vocalizations emitted by male bats sitting on display spots. Sonograms were created using a 1024 FFT and a Hamming Window with 75% overlap.

Our observations of individually identifiable males (*N* = 17) indicated high site fidelity. No male established a new display spot; only already existing spots were occupied. Fourteen males, identifiable through fur trims or scars, were faithful to their display spot throughout the entire season. From mid‐August to mid‐September, these males were consistently found at the same spot. The remaining three individually identifiable males disappeared from their roosts by the end of July, preventing us from conducting further observations during the mating season. Moreover, throughout the 2‐year study period, we observed several conspicuous males (*N* = 3), identifiable by distinctive characteristics such as a notably ruptured ear, returning to the same roost and to the same display spot in the following year. These anecdotal observations hint at long‐term fidelity and should be investigated in the future. Notably, there were no instances where two males were simultaneously observed at the same display spot. As soon as the males had established themselves at the spots and the number of males inside the roost remained stable, any attempt by one male to approach a display spot already occupied by another male resulted in aggressive behavior (*N* = 37; we noted aggressive behaviors in 100% of the attempts). Aggression included hitting with closed wings, biting, fast and targeted crawling toward the other bat, accompanied by emitting vocalizations (Figure 4B; the corresponding recording is provided in the Supporting Information). Apart from the conspicuous aggressive and territorial behaviors during the time when the occupancy of the display spot was sorted out, we did not observe any other conspicuous male–male interactive behaviors.

However, we consistently recorded the emission of various vocalizations inside the male roost (Figure 4C), suggesting social interactions among the male bats or courtship behavior toward female bats. For two roosts, long‐term acoustic monitoring was conducted from late May/early June to October 2023 (total recording hours = 2794). The number of trills varied significantly across the season, showing both increasing trends and curvilinear seasonal patterns (Figure 2C,D). Starting from the end of May/beginning of June (the beginning of the season), the number of trills shows a strong linear increase (estimate = 2.2733, *p* < 0.001), followed by a peak in August (quartic effect; estimate = −0.6366, *p* < 0.001), culminating in a significant reduction of trills by the end of September (fifth‐order polynomial term; estimate = −0.1611, *p* = 0.032). This suggests that trill‐like vocalizations follow a clear seasonal pattern, peaking mid‐season during August and diminishing toward the season's end in October (Table S3). These findings were consistent with our field observations, where we also noted a vocalization peak in August, and corresponded to the pattern of male roost occupancy.

**FIGURE 5:**
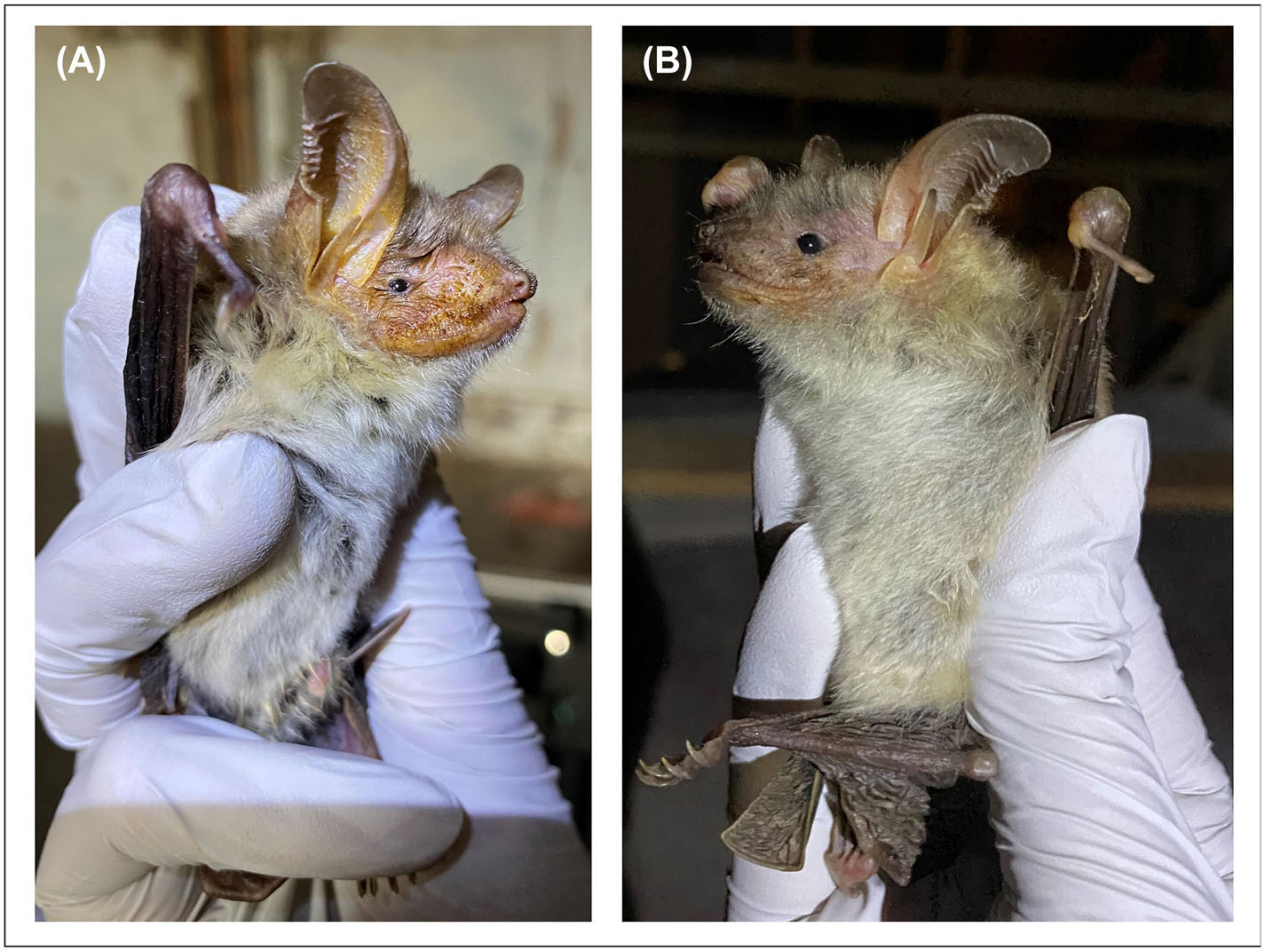
Temporal changes in facial secretion in male bats. The photographs document the amount of facial secretion on two individual male bats in (A) July and (B) September. In July, the yellowish or brownish secretion was prominent. By the end of September, we observed a noticeable reduction and a near absence of facial secretion.

Furthermore, we observed a notable yellow secretion on the males’ faces during our study period. The visible fluid secretion wet the whole face up to the ears. Once the secretion dried, it left a yellowish or brownish‐encrusted appearance. The secretion was predominantly visible in the months of July and August (Figure 5). By mid to late September, the intensity of the yellow secretion gradually diminished, indicating a temporal change in its presence (Figure 3); interestingly, the predominant presence of the yellow secretion coincided with the mating season. Males were never observed to mark females or display spots with their facial secretion. We were unable to determine for what purpose the yellow secretion might be used in the context of mate selection.

### Mating behavior

From mid‐August to mid‐September, we observed females arriving at the male roosts (Figure 3) and noted an increase in mating events. Mating events were observed from August 17th until September 15th, with a total of 27 observed mating events in three different roosts. We did not observe any instances of forced mating events. Upon inspection, we frequently encountered pairs consisting of one female and one male at the display spots (*N* = 55). In 18 instances, we observed two females sitting alongside a single male, although copulations never occurred while both females remained present. Notably, our observations revealed a very targeted female mate choice behavior, characterized by their direct flight toward a specific male and landing in close proximity to him, without interacting with the other males in the same roost prior to the female's decision.

**FIGURE 6:**
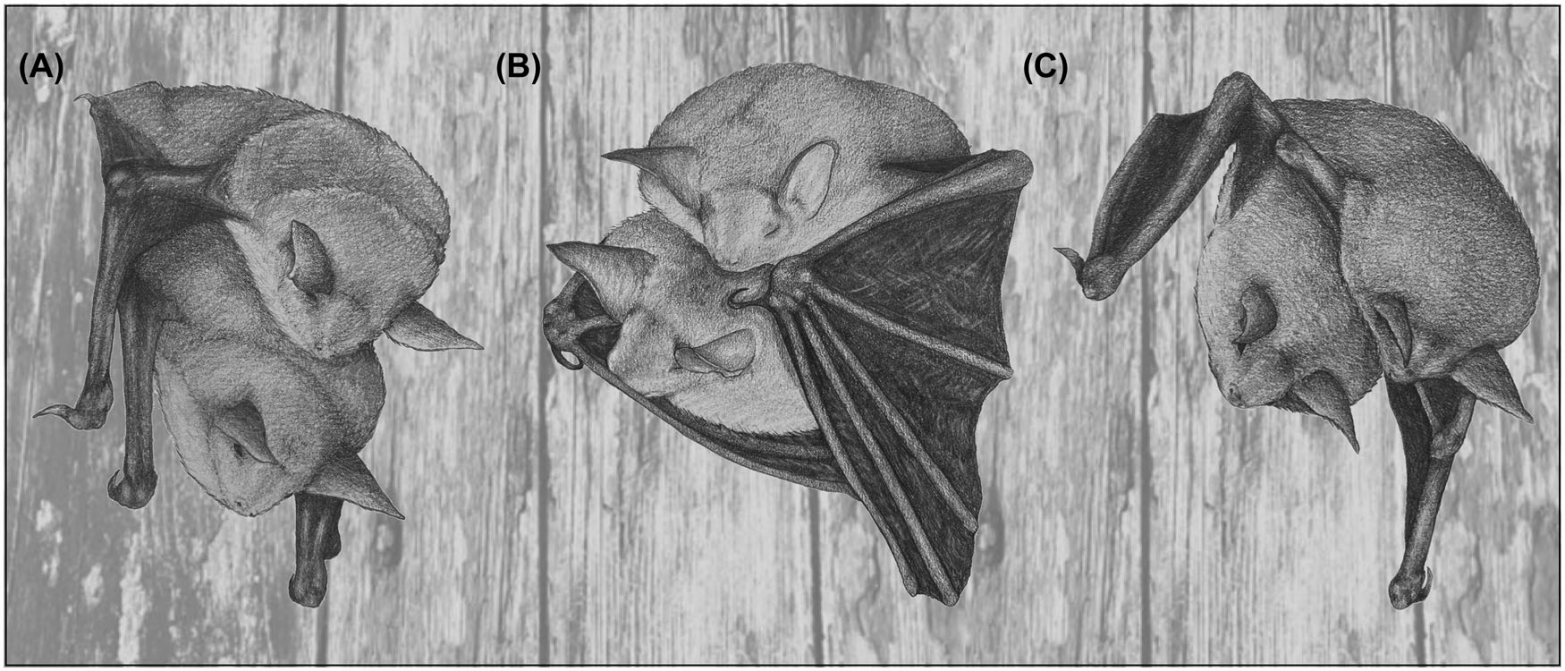
Noticeable postures of *M. myotis* during mating. (A) Posture during copulation. The male roosts with its belly on the back of the female. This posture is often accompanied by aggressive vocalizations. (B) The male encloses the female completely with its wings. During this posture, both individuals stay calm and do not vocalize. (C) The male resting on the back of the female, with the female slightly opening her wings. In this posture, males occasionally emitted social vocalizations. All postures are strictly related to mating and were observed exclusively in this context. Drawings were made by Emma Dittrich.

Observations of the copulation behavior of *M. myotis* revealed a series of distinct behaviors and body postures. When a female chose her mate and landed next to him, both individuals remained calm and quiet for extended periods, ranging from 25 min to 3 h and 10 min (mean = 1 h 58 min; SD = 32.8 min), before the copulation attempt proceeded. The copulation itself was initiated either by the female crawling underneath the male (*N* = 9) or the male crawling onto the female's back (*N* = 18). After the male was on the back of the female, the female started struggling and vocalized during her attempts to crawl away. The male responded by restraining the female with his closed wings, pushing her underneath him and by licking and biting the neck area of the female. During this behavior, the male copulated by moving his pelvis rhythmically for a few seconds (Figure 6A). Following the copulation, the female became calm again, the male loosened his restraining posture and both bats groomed themselves. This was followed by a noticeable body posture, where the male opened its wings and wrapped them completely around the female, with both individuals remaining calm (Figure 6B; video sequences are provided in the Supporting Information). In some cases, both bats closed their eyes and rested together for durations ranging from 50 min to 2 h and 25 min (mean = 1 h 11 min; SD = 19.77 min). During this body posture, we did not record any vocalizations. Afterward, the male opened its wings again and released the female.

**FIGURE 7:**
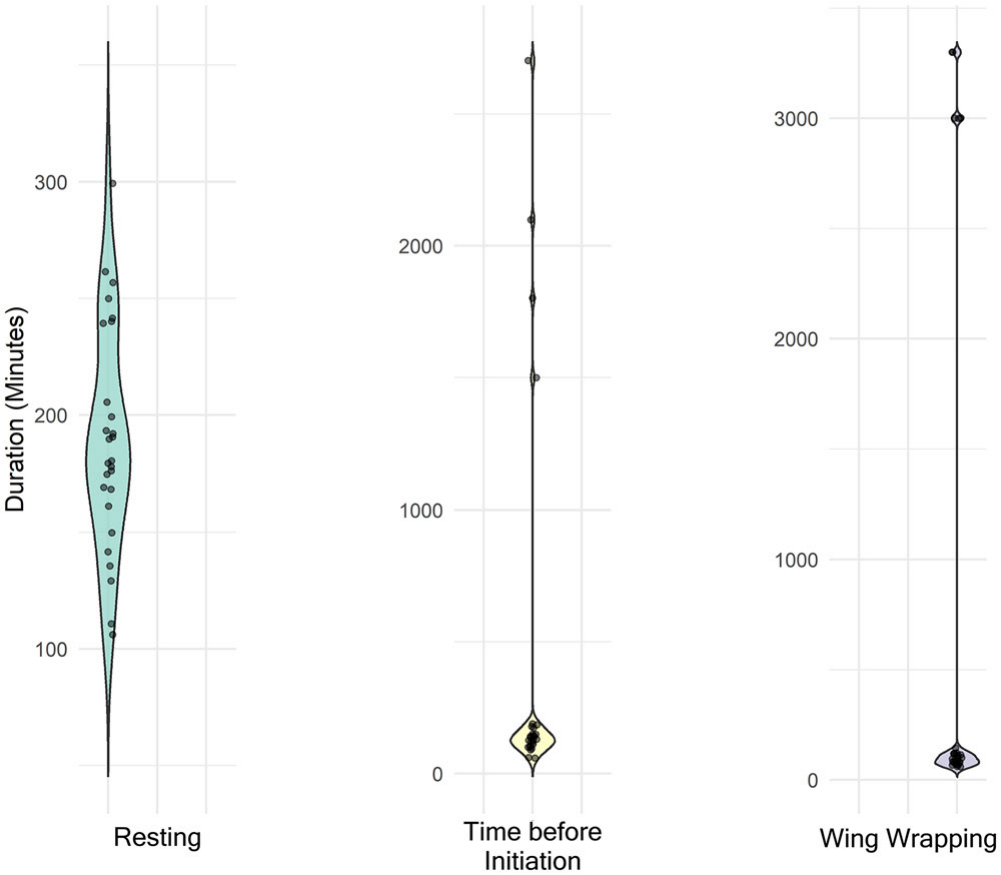
Duration of behavioral patterns in 27 observed mating events. *Resting*: Post‐copulation; the male either crawls on the female's back or both partners rest side by side on the display spot. *Time before Initiation*: Duration between female arrival to the initiation of mating. *Wing Wrapping*: Wing wrapping refers to the duration during which the female is enveloped by the male's wings. These positions are cycled and repeated throughout each mating event.

In some instances, after the male opened its wings, the male and female continued to rest next to each other. Alternatively, the male crawled back onto the female's back. However, this behavior did not appear to be for copulation, but rather for the male to rest on the female's back. During this period, the female slightly opened her wings, supposedly to support the male, and intermittently licked the mouth area of the male (Figure 6C). This posture was always followed by another copulation. On average, a pair copulated four times (mean = 3.96, ranging from two to six times, SD = 1.04) and did not leave their spot during a mating event. The longest mating event we were able to monitor without interruption (i.e., the male and female lingered at the male's roosting spot) lasted 34 h. The observed postures and behaviors during a mating event can be repeated multiple times with varying durations (Figure 7).

After a mating event, the female left the male roosting spot again without mating with another male from the same roost. However, because females were not marked, we do not know whether the same female returned to mate with the same male again or with another male belonging to the same roost.

## DISCUSSION

Our 2‐year study of *M. myotis* in six roosts provides substantial insights into male roost occupancy, mating behaviors, territoriality, and phenology, highlighting a phenological cycle in the male behavior of *M. myotis*.^38^, ^41^ We observed a distinct seasonal pattern throughout the season. At the beginning of the season, in late May and June, the number of males inside the roosts fluctuated, and they did not remain faithful to a single display spot. During this period, trill vocalizations were rare, and no yellow facial secretion was observed. By late July and early August, the number of males had stabilized, and display spot occupation remained stable. This suggests strong territorial claims, which were also reflected in increased aggressive behavior toward other males. During this time, trill‐like vocalizations became frequent, and yellow facial secretions on the males’ faces were prominent. The peak of trill‐like vocalizations occurred in August, aligning with the onset of the mating season. From mid‐August to mid‐September, females arrived at male display spots, and mating events were observed. Throughout this period, again, display spot occupation remained stable. By late September, males gradually left the roost, the yellow secretion diminished, and only a few trill‐like vocalizations were recorded. By mid‐October, all roosts were vacant.

Our findings on male display spot fidelity are in accordance with prior findings. Despite the impact of male display activity and aggression, lek attendance remains a key factor for male mating success in many species.^19^ Zahn and Dippel^38^ found that male *M. myotis* fidelity to preferred sites increases from May to August, peaking during the August–September mating season. Additionally, McCracken and Wilkinson^7^ state that some males use the same spot for up to 6 years, a pattern of long‐term fidelity we also observed over the course of our study. Our findings underscore the importance of specific display spots for mating success, supporting our assumption that established territories play a crucial role in the reproductive strategy of male *M. myotis*.

### Mating system

To date, the mating system of *M. myotis* has not been categorized and seemed to not fit neatly into any existing category.^38^ However, our observations suggest that *M. myotis* exhibits a lek mating system. Lekking can be defined by four characteristics: (1) males aggregating to display within a specific location; (2) females do not benefit from any resources provided by the males; (3) males provide no parental care to the offspring; and (4) females can choose freely among the males.^3^, ^5^, ^15^ We observed aggregating males exhibiting territorial behavior against other males during the mating season of *M. myotis*. This was also mentioned by Zahn and Dippel,^38^ who found aggressive behavior between males but not against females. Moreover, Zahn and Dippel^38^ described five vocalizations, including chirping, which closely resemble the trill‐like vocalizations we observed within the roosts. While they were unable to determine the specific purpose of this vocalization and found no special behaviors exhibited by males to attract females, we propose that these specific vocalizations likely play a role in mate attraction and are also crucial for male spacing. In mammals, acoustic signaling in the context of mating has been found in various taxa, for example, red deer stags (*Cervus elaphus*).^46^ This is also true for bats, as many species use vocalizations to attract mates.^26^, ^30^, ^47^ The vocal repertoire of bats can be complex and some bat species use specific vocalizations solely in the context of mating (e.g., *Tadarida brasiliensis* ^32^ and *Pipistrellus nathusii* ^48^), thus those vocalizations can be limited to the mating season. This pattern also applies to *M. myotis*.

The strongly stained display spots may further function as olfactory cues for the females, as suggested for other bat species.^49^ Olfactory cues play a crucial role in mate selection, as many animal taxa rely on chemical signals during mate choice due to their reliability as honest signals.^50^ Chemical cues are particularly well‐suited for this purpose since they are unintentionally produced during daily activities, making them difficult to fake. Individual odors enable potential mates to distinguish between individuals and gather valuable information, such as social status^51^ or group affiliation.^52^, ^53^ Thus, this might also be one important component of the multimodal display of *M. myotis*.

Multimodal displays are common in lekking species,^3^ as males must advertise effectively in order to attract females while defending territories. For example, male *Hypsignathus monstrosus* bats produce low‐frequency vocalizations accompanied by wing flapping during mating displays;^30^ a multimodal display consisting of visual and vocal components. In *M. myotis*, we observed a combination of vocal and olfactory displays, further supporting the presence of a lek mating system. Consistent with Zahn and Dippel,^38^ we found no evidence that males provide resources to females. Male parental care is largely absent in bats,^7^, ^54^ and this also includes *M. myotis*. Similar to the observations of Zahn and Dippel,^38^ females moved freely within the roosts and among males, reinforcing the lek mating hypothesis. Similar male aggregation and small territory occupation were reported in *M. myotis* populations in Portuguese caves,^34^, ^55^ aligning with our findings.

While knowledge of lek breeding in temperate bat species remains limited, its prevalence may be higher than currently recognized.^15^ However, studying bat mating systems is challenging, which may contribute to the perceived rarity of lekking behaviors.^15^ Nevertheless, understanding lek systems is valuable for behavioral ecology and conservation efforts. Identifying male display site requirements, sexually mature individual distributions, and female mate selection patterns is essential for effective conservation strategies.^56^

### Territorial behavior and aggressive vocalizations

Territorial displays of bats can be multimodal, including acoustic, visual, and olfactory signals,^57^ and can escalate into physical fights.^58^, ^59^ In gregarious species, aggressive behaviors influence individual status and mating success, with territorial males often achieving greater reproductive success than nonterritorial males.^60^, ^61^, ^62^, ^63^ Territoriality can arise from resource defense or lek defense, where female choice is influenced by male displays as indicators of mate quality.^21^, ^64^ Although there are several studies on aggressive behavior in bats,^58^, ^65^ specific observations for *M. myotis* have remained anecdotal.

Male bats often defend territories that provide essential resources for females,^5^ such as feeding ranges or roosting sites, a strategy known as resource‐defense polygyny. In contrast, mate‐defense polygyny occurs when males guard individual mating partners.^5^ However, in *M. myotis*, females receive no benefits beyond mating, ruling out resource‐defense polygyny. Furthermore, females are free to move and mate with multiple males, eliminating mate‐defense polygyny as an explanation for territorial behavior. Instead, males defend small display spots within the mating roost, supporting the conclusion that the territoriality of *M. myotis* is a behavioral trait of a lek mating system. We observed different aspects of territorial behavior in *M. myotis*, which was triggered by males attempting to intrude on already occupied spots. Male *M. myotis* establish and defend roosting sites of approximately 250 cm^3^ in area within attics,^7^ which may also be inhabited by females and juveniles. This is in accordance with our observations, where up to two females sat next to one male within the same display spot. Our observations align with lek mating traits, where males congregate at display sites and actively defend them.^5^ Notably, males never displayed aggression toward females, indicating that aggressive behavior is primarily used to establish and reinforce territorial boundaries through agonistic interactions with other males.

In *M. myotis*, aggressive behaviors involved actions such as hitting with closed wings, biting, and rapidly crawling toward the intruder, accompanied by vocalizations. Our observations suggest that there is multistage aggression, beginning with aggressive vocalizations and progressing to physical confrontations, including biting. During these agonistic interactions, we recorded distinct vocalizations, matching the descriptions by Walter and Schnitzler,^66^ who identified context‐specific vocalizations associated with aggressive behavior. In conclusion, our observations provide strong evidence for territorial behavior in *M. myotis*, including vocal and physical stages of aggression. The defense of display spots inside male roosts seems to be crucial for the mating success of males, thus the prior establishment of selected display spots and the defense of the latter is a key aspect in mating behavior. This behavior strongly supports our assumption that *M. myotis* has a lek mating system.

### Complex male vocalizations

We recorded conspicuous, trill‐like vocalizations inside male roosts and provide a preliminary insight into the complexity of vocalizations in a courtship and mating context for *M. myotis*. We recorded complex vocalizations emitted exclusively by males from July to September, which aligns with the species’ mating season. Notably, the vocalization activity of males increased in July and peaked in August, right before the disintegration of maternity colonies. This temporal pattern suggests a strategic increase in vocal display behavior. Social vocalizations are commonly used among various species of bats^20^ and are frequently utilized to mark male territories and directly attract females. However, to date, the role of vocal signals for female mate choice in bats has received comparatively little attention.^67^, ^68^

Complex vocalizations increased in July, indicating that males establish their display spots well before the mating season begins, since during this time, only males were present inside the roosts. In numerous animal species, including bats, territory defense involves the use of vocal signals,^2^ which helps in setting territory boundaries.^56^, ^69^, ^70^, ^71^, ^72^ Territorial vocalizations can also convey aspects of male quality and thus play a key role in female choice,^2^, ^21^ also in bats.^47^, ^59^ Studies focusing on various taxa have demonstrated that male vocalizations can trigger female phonotaxis,^73^, ^74^, ^75^ including in bats.^47^ Females of *M. myotis* flying by or roosting nearby might be attracted by calls that males direct at other males and extract valuable information about the sender. Thus, male vocalizations could function as an important communication tool, providing females a prior assessment of potential mates, which would suggest a dual function of complex vocalizations for rival deterrence and female attraction. However, further research is needed to determine the information encoded in these vocalizations.

### Facial secretion

We noted the presence of a conspicuous secretion on the faces of males, predominantly visible in July and August, with its intensity diminishing by mid to late September. The timing of the presence of this secretion aligns with the mating season. When potential mates are in close proximity, olfactory signals might have an effect on the course of interaction. Thus, the facial secretion might be used in mating contexts, potentially for scent marking and/or as a visual signal. The use of olfactory cues in mate choice has been confirmed in various animal taxa,^50^ including bats.^76^, ^77^ Secretions are used for scent‐marking conspecifics, territories, or themselves.^32^, ^78^, ^79^ In some species, the glands are only present in males and are active only during the mating season.^80^, ^81^ This aligns with our observations that the trait is only present in males inside the mating roosts. The secretion appeared only on the face, but we do not know whether it was sequestered there or transported there from glandular secretions elsewhere on the body. We did not observe males rubbing the secretion on females during mating, leading us to conclude that its function likely does not involve marking females. Instead, the facial secretion may be used for marking territories, displaying spots, or acting as an olfactory display for females, thus conveying various messages or information relevant to reproductive behavior. However, the exact purpose of this secretion remains unclear and warrants further investigation.

### Copulatory behavior

Once females entered a male roost, they flew directly toward a specific male and landed in close proximity to him without interacting with other males in the same roost, indicating a highly targeted mate choice. A prior study observed similar behavior and reported females actively visiting males at the male roosts.^38^ This suggests that females have specific criteria or preferences when selecting mates, but it is currently unclear how they assess males.

The mating process followed a series of distinct behaviors and body postures, part of which were already described in a prior study.^38^ Post‐copulation behaviors, such as the male wrapping his wings around the female, suggest that mate‐guarding behavior occurs in *M. myotis*. Male precopulatory and postcopulatory strategies have evolved to increase the reproductive success through various tactics,^11^ whereby males follow females and repel other males to ensure copulation exclusivity. In *M. myotis*, mating pairs separate again after several hours but females may remain with the same male for 2−3 days and even return to the same male several times for additional mating.^38^ Mate guarding can offer advantages to both sexes.^82^ For males, extended guarding might be beneficial for securing copulation opportunities and enhancing paternity success.^82^, ^83^ Additionally, repeated copulations enable males to transfer more sperm, favoring both fertilization chances and paternity success,^84^, ^85^ which can ultimately outweigh the potential costs associated with guarding against males. However, females in *M. myotis* appeared to be free to leave the male and were not forced to remain on the display spot by the male, suggesting that guarding is also advantageous for the females. Studies in other taxa have shown that mate guarding can benefit females by reducing predation risk for females.^82^, ^86^ Additionally, by staying with a particular male, females have more time for male assessment and can eventually influence the paternity of a preferred male through multiple copulations, resulting in increased sperm transfer.^82^ Thus, the observed guarding behavior likely provides benefits to both sexes.

Another conspicuous posture observed was the male resting on the female's back. During this time, the female licked the male's face, a behavior consistently followed by copulation. This interaction, along with the male's resting posture, may facilitate repeated mating events. In temperate‐zone bats, including those in the Vespertilionidae family, females store sperm in their reproductive tract through winter, with fertilization delayed until spring.^29^, ^87^, ^88^ This unique reproductive strategy increases sperm competition among males and may explain both the repeated copulations and mate‐guarding behavior observed. For *M. myotis*, males enter winter with minimal to no cauda‐epididymal sperm reserves, making late‐summer mating critical to maximize their chances of paternity.^89^

Notably, we observed that mating events often involved multiple copulations, with pairs remaining at the display site for several hours. During this time, behaviors such as resting together, additional grooming, and intermittent licking by the female frequently preceded further copulations. Repeated copulations may also be advantageous for females, especially if a single mating is insufficient to ensure fertilization or in terms of female choice.^90^

The observed behaviors emphasize the complexity of the *M. myotis* mating system, where female choice and male territoriality play important roles. The presence of females and the high number of mating events during the late summer months align with the peak in male roost occupancy noted earlier.

## CONCLUSIONS

Males exhibited strong territorial behavior, maintaining exclusive use of display spots and engaging in aggressive interactions to defend these areas. This territoriality likely plays a crucial role in attracting and securing mating opportunities as females may select mates based on specific criteria, such as a male's ability to successfully establish and defend a territory. This study provides valuable insights into the mating system, phenology, and territoriality of *M. myotis* and emphasizes the species’ reliance on well‐defined and stable display sites. Understanding the intricacies of mating systems offers critical information about habitat and resource requirements, directly informing conservation efforts.^91^

Our results highlight the importance of incorporating phenology in shaping conservation strategies, particularly in the timing of roost restoration. Current roost conservation practices often assume that male *M. myotis* are more flexible than females, allowing for renovations in male roosts without seasonal restrictions. However, our results challenge this assumption, revealing that males show strong, long‐term fidelity to traditional display spots, with no evidence of establishing new sites. This aligns with anecdotal observations that some male roosts have persisted for decades. To ensure the effectiveness of conservation efforts, it is essential to prioritize the preservation of existing display spots or, at a minimum, replicate them at the same locations during and after any modifications of the male roosts. Furthermore, restoration activities should be carefully timed not only for nursery roosts—where seasonal restrictions are already well established—but also for mating roosts to minimize disruptions in alignment with the distinct phenological patterns observed in males. These results and considerations are essential for both fundamental research and effective, evidence‐based conservation strategies.

## AUTHOR CONTRIBUTIONS

L.P. and M.K. designed the study. L.P. collected the data. A.L. assisted with the data collection. L.P. and M.N. analyzed the data. M.K. supervised the study. L.P. wrote the initial manuscript. All authors contributed to the final version of the manuscript.

## COMPETING INTERESTS

The authors declare that they have no competing interests.

## PEER REVIEW

The peer review history for this article is available at: https://publons.com/publon/10.1111/nyas.15390.

## Supporting information



Supporting Information

Supporting Information

Supporting Information

## Data Availability

The data that support the findings of this study are available from the corresponding author upon request.
