# Supplementary material for: Mating system and copulatory behavior of the greater mouse‐eared bat (Myotis myotis)
Source: Ann N Y Acad Sci. 2025 Jun 18;1549(1):185–98. doi: 10.1111/nyas.15390 (PMC12309444; doi:10.1111/nyas.15390)
Supplement: Supplementary file 1 — Supporting Information [file NYAS-1549-185-s002.docx]

**Table S1:** Total number of inspection days and observation time [hours] for every roost during the study period.

| **Roost ID** | **2022** | | | **2023** | | |
| --- | --- | --- | --- | --- | --- | --- |
|  | Inspection days | In-person observation hours | Maximum number of males inside the roost | Inspection days | In-person observation hours | Maximum number of males inside the roost |
| **1** | 23 | 75.8 | 7 | 20 | 87.5 | 6 |
| **2** | 23 | 77.5 | 20 | 27 | 141.0 | 21 |
| **3** | 22 | 67.0 | 7 | 26 | 93.8 | 8 |
| **4** | - | - | - | 20 | 90.5 | 13 |
| **5** | - | - | - | 18 | 57.1 | 6 |
| **6** | - | - | - | 16 | 65.3 | 9 |

**Table S2:** Statistical results of the analysis of male occupancy throughout the season.

|  | **Parameter** | **Estimate** | **Std. Error** | **z-value** | **p (>\|z\|)** |
| --- | --- | --- | --- | --- | --- |
| **Model 1: Number of male bats over the season** | | | | | |
| GLMM  Poisson-distribution | *(Intercept)* | 1.248121 | 0.201299 | 6.200 | 5.63e^-10^ *** |
| AIC 865.4 | Linear Trend | 0.663770 | 0.201078 | 3.301 | 0.000963 *** |
|  | Quadratic Trend | -2.116010 | 0.183974 | -11.502 | < 2e^-16^ *** |
|  | Cubic Trend | 0.006698 | 0.134968 | 0.050 | 0.960419 |
|  | Month ^4^ | -0.342201 | 0.087678 | -3.903 | 9.50e^-05^ *** |
|  | Month ^5^ | -0.021116 | 0.059871 | -0.353 | 0.724323 |
| **Post-hoc analysis** | Estimated marginal means + Tukey method | | | | |
|  | **Parameter** | **Estimate** | **Std. Error** | **z-ratio** | **p (>\|z\|)** |
|  | May – Jun | -1.8006 | 0.313 | -5.755 | < 0.0001 |
|  | May – Jul | -2.347 | 0.307 | -7.637 | < 0.0001 |
|  | May – Aug | -2.4751 | 0.306 | -8.095 | < 0.0001 |
|  | May – Sep | -2.2829 | 0.306 | -7.46 | < 0.0001 |
|  | May – Oct | -0.7957 | 0.331 | -2.404 | 0.1548 |
|  | Jun – Jul | -0.5464 | 0.101 | -5.431 | < 0.0001 |
|  | Jun – Aug | -0.6745 | 0.0951 | -7.092 | < 0.0001 |
|  | Jun – Sep | -0.4823 | 0.0962 | -5.015 | < 0.0001 |
|  | Jun – Oct | 1.0049 | 0.159 | 6.321 | < 0.0001 |
|  | Jul – Aug | -0.1281 | 0.0751 | -1.705 | 0.5283 |
|  | Jul – Sep | 0.0641 | 0.0766 | 0.837 | 0.9607 |
|  | Jul – Oct | 1.5513 | 0.148 | 10.486 | < 0.0001 |
|  | Aug – Sep | 0.1922 | 0.0687 | 2.796 | 0.0581 |
|  | Aug – Oct | 1.6794 | 0.144 | 11.653 | < 0.0001 |
|  | Sep – Oct | 1.4873 | 0.145 | 10.271 | < 0.0001 |
| Results are given on the log (not the response) scale. | | | | | |

**Table S3:** Statistical results of the analysis of the distribution of trill-like vocalizations.

|  | **Parameter** | **Estimate** | **Std. Error** | **z-value** | **p (>\|z\|)** |
| --- | --- | --- | --- | --- | --- |
| **Model 2: Number of trill-like vocalizations** | | | | | |
| Negative binomial regression model | *(Intercept)* | -1.22753 | 0.12789 | -9.598 | < 2e^-16^ *** |
| AIC 13620 | Linear Trend | 2.27332 | 0.43933 | 5.174 | 2.29e^-07^ *** |
|  | Quadratic Trend | -2.33160 | 0.39918 | -5.841 | 5.19e^-09^ *** |
|  | Cubic Trend | 0.52508 | 0.28227 | 1.860 | 0.0629 . |
|  | Month ^4^ | -0.63655 | 0.16166 | -3.938 | 8.23e^-05^ *** |
|  | Month ^5^ | -0.16107 | 0.07521 | -2.142 | 0.0322 * |
| **Post-hoc analysis** | Estimated marginal means + Tukey method | | | | |
|  | **Parameter** | **Estimate** | **Std. Error** | **z-ratio** | **p (>\|z\|)** |
|  | May - Jun | -2.9598 | 0.732 | -4.045 | 0.0007 |
|  | May - Jul | -3.6997 | 0.722 | -5.123 | <0.0001 |
|  | May - Aug | -3.7271 | 0.722 | -5.165 | <0.0001 |
|  | May - Sep | -4.1436 | 0.722 | -5.74 | <0.0001 |
|  | May - Oct | -3.0882 | 0.73 | -4.231 | 0.0003 |
|  | Jun - Jul | -0.7399 | 0.142 | -5.225 | <0.0001 |
|  | Jun - Aug | -0.7673 | 0.138 | -5.544 | <0.0001 |
|  | Jun - Sep | -1.1838 | 0.141 | -8.385 | <0.0001 |
|  | Jun - Oct | -0.1284 | 0.177 | -0.727 | 0.9787 |
|  | Jul - Aug | -0.0274 | 0.0629 | -0.435 | 0.998 |
|  | Jul - Sep | -0.4439 | 0.0731 | -6.072 | <0.0001 |
|  | Jul - Oct | 0.6115 | 0.126 | 4.837 | <0.0001 |
|  | Aug - Sep | -0.4165 | 0.0664 | -6.273 | <0.0001 |
|  | Aug - Oct | 0.6389 | 0.122 | 5.226 | <0.0001 |
|  | Sep - Oct | 1.0554 | 0.128 | 8.227 | <0.0001 |
| Results are given on the log (not the response) scale. | | | | | |
